# Back Pain among COVID-19 Positive Health Care Workers in a Tertiary Care Hospital in Nepal: A Descriptive Cross-sectional Study

**DOI:** 10.31729/jnma.7084

**Published:** 2021-10-31

**Authors:** Shriraj Shrestha, Ayush Bajracharya, Saurav Dahal, Parash Bhandari, Basanta Maharjan, Suraj Bajracharya

**Affiliations:** 1 Department of Orthopaedics and Trauma, KIST Medicai College and Teaching Hospital, Laiitpur, Nepal

**Keywords:** *back pain*, *COVID-19*, *health care workers*

## Abstract

**Introduction:**

The world has been threatened with the emergence of the Novel Corona Virus straining the health care system and creating a global pandemic. This is not the first pandemic, and it certainly will not be the last to affect humanity. As the medical community is exposed to these highly contagious new diseases with arrays of symptoms like fever, cough, shortness of breath, anosmia, insomnia, and myalgia. Back pain can also be considered as one of the symptoms of COVID-19 infection. Therefore, this study aimed to find out the prevalence of back pain among the Health care workers who were tested positive for COVID-19 by the end of their isolation period.

**Methods:**

This descriptive cross-sectional study was done from April 2021 to June 2021 in KIST Medical College and Teaching Hospital, Imadol, Lalitpur, Nepal, after receiving ethical approval from the Institutional Review Committee (Registration number: 2077/078/57). Convenience sampling was done. Data collection and entry were done in Microsoft excel, point estimate at 95% Confidence Interval was calculated along with frequency and proportion for binary data.

**Results:**

Out of 156 COVID-19 positive patients, the prevalence of back pain was seen among 64 (41%) patients (95% Confidence Interval= 42.23-57.75). Among them 21 (32.8%) were males and 43 (67.2%) were females. Likewise, the minimum age was 20 years and the maximum was 68 years with a mean of 33.5±10.28.

**Conclusions:**

This study demonstrated that a high proportion of healthcare workers were suffering from back pain and the findings are comparable to the data from other international studies.

## INTRODUCTION

The Coronavirus Disease (COVID-19) caused by the severe acute respiratory syndrome coronavirus 2 (SARS-CoV-2) has challenged the health system worldwide and created a global pandemic.^1^ Health care workers (HCWs) are at high risk as they are regularly exposed to the disease and due to their prolong duty hours with extra protective gears may suffer from back pain.^2^ Coronaviruses are RNA-positive viruses with spike-like projections, with diameters ranging from 60 to 140nm on their top, thus providing a crown-like appearance under an electron microscope.^3^

There are spectrum of severity in COVID-19 patient widely from asymptomatic to critical with majority being only mild symptomatic.^4^ In our clinical practice, patients suffering from COVID-19 often complain of back pain and to the best of our knowledge, there is no such study conducted in Nepal.

This study aimed to find out the prevalence of back pain among the Health care workers who were tested positive for Coronavirus disease 2019 (COVID-19) by the end of their isolation period.

## METHODS

This was a descriptive cross-sectional study conducted from April 2021 to June 2021 in KIST Medical College and Teaching Hospital, Imadol, Lalitpur, Nepal. The study was carried out after receiving ethical approval from the Institutional Review Committee (Registration number: 2077/078/57). The study was done among HCWs including doctors, nurses and paramedics (health assistants, community medical assistants, lab technicians and pharmacist), hospital attendents and the administrative staff. All the HCWs who were tested positive for COVID-19 with Polymerase Chain Reaction (PCR) and gave consent were included in the study. Exclusion criteria of the study were those who had chronic back pain (more than three months) and who did not give written consent. Convenience sampling was done and the sample size was calculated using the formula,

n = Z^2^ × p × q / e^2^

  = (1.96)^2^ × 0.5 × (1-0.5) / (0.08)^2^

  = 150

Where,

n = minimum required sample size
Z = 1.96 at 95% Confidence Interval (CI)
p = prevalence taken as 50% for maximum sample size
q = 1-p
e = margin of error, 8%

The required sample size was 150, however, 156 responses were included in the study. This data was retrieved from the hospital database. All data were collected after informing participants about the study objectives and significance of the study.

Once the written consent was taken, data were collected using the structured questionnaire which included detailed information regarding sociodemographic characteristics, like sex, age, marital status. Different occupational attributes, like working hours per day, department and physical activities were also noted. Those who had back pain during and after being COVID-19 positive were further asked to locate the site of pain like neck, shoulder, thoracic area, or lower back. The participants also needed to grade the severity of pain using the Visual Analog Scale (VAS) scoring system, during, and after the isolation period. It was pre-defined that patients with VAS pain scores of 0 had no pain, 1 mm to 30 mm were categorized as having mild pain, those with scores of 31 mm to 69 mm were categorized as having moderate pain, likewise 70 mm to 99 mm as severe pain and 100 mm as extreme pain.^5^

All data were analyzed by Statistical Package for the Social Sciences (SPSS) version 16 using appropriate statistical tools. Numerical data were analyzed using frequency, mean and standard deviation. Point estimate at 95% confidence interval was calculated along with frequency and percentage for binary data.

## RESULTS

Among the 156 COVID-19 positive patients 64 (41%) (95% Confidence Interval= 42.23-57.75) of them developed back pain, out of which 21 (32.8%) were male and 43 (67.2%) were female. Likewise, the minimum age was 20 years and the maximum was 68 years with a M ean±SD, 33.5±10. 28. Back pain among the COVID-19 positive patients was more prevalent among the doctors 24 (37.5%), followed by nurses and paramedics 21 (32.8%), housekeeping staff 11 (17.2%), and administration staff 8 (12.5%). Most of the infected staff with back pain were married 41 (64.1%).

**Table 1:** Different sites of back pain among COVID positive patients (n = 64).

Back pain site	n (%)
Neck	24 (37.5)
Shoulder	15 (23.4)
Thorax	41 (64.1)
Low back	52 (81.2)

**Figure 1:**
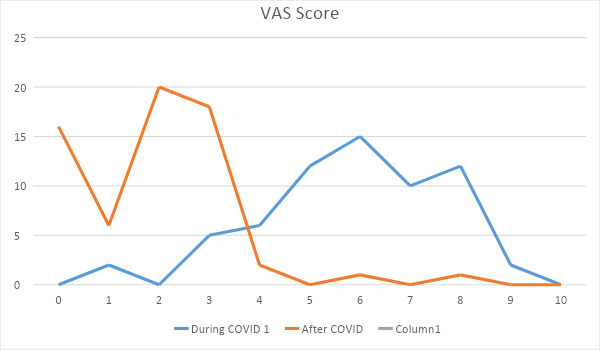
VAS during and after being infected with COVID (n= 64).

According to the VAS score, 7 (10.9%) patients had mild back pain, 33 (51.6%) had moderate back pain and 24 (37.5%) had severe back pain during the time of COVID-19 infection, which later on ( after 14 days) decreased to no pain in 16 (25%) patients, mild in 44 (68.7%), moderate in 3 (4.7%) and severe in only one (1.6%) patient.

## DISCUSSION

Health care workers (HCWs) are exposed to infected patients for a long time and work overload with minimum rest during the time of COVID-19 place them at a high risk category. Only a few studies regarding COVID-19 with back pain have been conducted worldwide. However, there has not been such a study conducted in Nepal during the COVID-19 pandemic, so we have tried to find out the prevalence of back pain among the COVID-19 positive HCWs in a tertiary care hospital in Nepal. There is a wide array of symptoms for COVID-19 which are often nonspecific, but the identification of the symptom for early detection of the disease is crucial.^6^ Around 80% of the patient with COVID-19 have mild symptoms and only the remaining 20% requires intervention.^7^

In our study, the prevalence of back pain among the COVID-19 positive HCWs in a tertiary care hospital who stayed in isolation was 41%. This was similar to a study conducted by Murat S et al in Istanbul Medeniyet University which showed 36.8% of their admitted patient diagnosed with COVID-19 to have back pain.^8^ In the same study male to the female percentage of back pain in COVID-19 positive patients were almost equal i.e. 46.6% and 53.3% respectively which was in contrast to our study which showed a female predominance of 67.2% as compared to male 32.8%. But, another study of COVID-19 patients conducted by Alkheraiji A, et al. showed a similar female predominance of 179 (63.7%) patients among 281 to have low back pain.^9^ A study conducted by Cuma Uz, et al. found half of their study population (50.5%) to have back pain during the time of COVID-19. They also constituted that patients with back pain had significantly higher pneumonia rates compared to those without.^10^ These studies were in concordant with the clinical data showing that 15% of patients with pneumonia had back pain.^11^ Another study carried out by Davis K found an association between COVID-19 pneumonia with severe back pain, he also observed cough and mild shortness of breath in patient with back pain.^7^

In our study 81.2% patients had lower back pain, which was very high as compared to a study conducted by Murat S et al which showed only 44 (33.1%) patients to have low back pain among a total of 210 admitted COVID-19 patients.^8^ Similar study conducted in the kingdom of Saudi Arabia showed a high incidence of low back pain among COVID-19 positive patients which was 79% and was close to our study.^9^ Another study conducted by Sagat P showed an increase in the number of low back pain from 38.8% to 43.8% among the citizen of Saudi Arabia during the period of quarantine.^12^ We further divided our patients with back pain into thoracic pain 64.1%, neck pain 37.5%, and shoulder blade pain 23.4% as shown in Table 1.

According to the VAS score, 10.9% patients had mild back pain, 51.6% had moderate back pain, and 37.5% had severe back pain during the isolation period. This showed that the moderate type of pain was observed more in COVID-19 patients with back pain which was in contrast to the study conducted by Alkheraiji A et al which showed 25.3% of their study population to have moderate type of back pain during the time of COVID-19. In another study carried out by Murat S et al found moderate type of pain to be in maximum number.^8^ In our study, a VAS score of 6 was perceived in 23.4% patients which were maximum in number.

During the time of isolation lack of physical exercise could be one of the reasons in our study population to have back pain. A study conducted by Crisafulli et al. has shown physical activity to have therapeutic and protective effects on the cardiovascular, pulmonary, musculoskeletal, neuro logical, immune, and endocrine systems even during the time of COVID-19.^13^ One more study carried out by Sahin et al. stated that the severity of back and neck pain during the time of COVID-19 infection increased as compared to the pre-infection period.^14^

At the time of our study, not all the PCR-positive patients were admitted to the hospital, only those patients who developed moderate to severe respiratory symptoms were admitted. Thus in our study, only 7.8% patients required hospitalization. Non of them were admitted due to severe back pain, rather all of them required intravenous medicine and oxygen support. Opposing to our study carried out in Istanbul showed pain to be one of the most common complaints for admitting the patient to hospital with COVID-19 which accounted nearly half of their admitted patients.^8^ As the symptoms of this disease can begin mildly, it can progress and lead to mortality, therefore it is necessary to have early diagnostic and treatment.

This study was done in a single institute with a limited sample size among the HCWs. Thus the findings may not be generalised. Further studies must be done in a larger sample size.

## CONCLUSIONS

This study demonstrated that a high proportion of healthcare workers were suffering from back painand the findings are comparable to the data from other international studies. Coronavirus is a new disease to mankind with different presentations, we as HCWs will have to deal with this disease in the months to come. As back pain could be one of the presenting symptoms of a patient visiting the Out Patient Department (OPD), the examining and attending HCWs must take proper precaution with necessary social distancing to avoid nosocomial transmission of the virus. Therefore it may be imperative to implement back pain as one of the symptoms of COVID-19 for early diagnosis of the disease to mitigate further devastation.
